# Psychosocial stress exacerbates doxorubicin-induced cardiotoxicity in adult C57BL/6N mice

**DOI:** 10.21203/rs.3.rs-8491866/v1

**Published:** 2026-01-08

**Authors:** Mary R. Daniel, Marianne K.O. Grant, Maria Razzoli, Juan E. Abrahante, Mohamed S. Dabour, Fernando Souza-Neto, Jop H. Berlo, Alessandro Bartolomucci, Beshay N. Zordoky

**Affiliations:** University of Minnesota; University of Minnesota; University of Minnesota; University of Minnesota; University of Minnesota; University of Minnesota; University of Minnesota; University of Minnesota; University of Minnesota

**Keywords:** Cardiotoxicity, Chronic Subordination Stress, Anthracyclines, RNA sequencing

## Abstract

Psychosocial stress is an established cardiovascular risk factor, yet its influence on chemotherapy-induced cardiotoxicity remains poorly understood. Doxorubicin (DOX), a widely used chemotherapeutic agent, is known to induce cardiotoxicity. However, whether concurrent psychosocial stress exacerbates this effect is unclear. This study aimed to determine the impact of chronic subordination stress (CSS) on DOX-induced cardiotoxicity using a clinically relevant ‘two-hit’ mouse model. Twelve-week-old male C57BL/6N mice were subjected to CSS for 26 days. DOX (8 mg/kg/week) or vehicle was administered during the final 3 weeks of CSS. Cardiac function was evaluated using echocardiography, while myocardial fibrosis was assessed histologically. Bulk RNA sequencing was conducted to identify differentially expressed genes (DEGs), with key genes validated by real-time PCR. Neither CSS nor DOX alone induced significant cardiac dysfunction. However, the combination of CSS and DOX led to both systolic and diastolic dysfunction, myocardial fibrosis, and increased mortality. Expression of cardiac stress markers *Nppa* and *Nppb* was significantly elevated by DOX, with CSS further amplifying *Nppa* expression. RNA sequencing revealed upregulation of pro-fibrotic genes (*Lgals3, Sprr1a*) and the pro-inflammatory cytokine *Il6* under combined CSS and DOX exposure. Gene set enrichment analysis showed dysregulation in metabolic, inflammatory, and cell cycle-related pathways. Psychosocial stress significantly worsens DOX-induced cardiotoxicity by promoting cardiac dysfunction, fibrosis, and maladaptive gene expression. This study highlights psychosocial stress as a critical risk factor for adverse cardiovascular outcomes in cancer patients receiving potentially cardiotoxic chemotherapy.

## INTRODUCTION

Cardiotoxicity is a well-recognized side effect of many chemotherapeutic agents. It can manifest as acute or chronic cardiac dysfunction, potentially leading to heart failure and other cardiovascular complications [1]. Out of all the chemotherapeutic agents, anthracyclines such as doxorubicin (DOX) are employed in the treatment of a wide variety of solid organ tumors and hematologic malignancies [2]. While an effective chemotherapeutic agent, DOX causes cumulative and dose-dependent cardiotoxicity, ranging from occult changes in myocardial structure and function to severe cardiomyopathy and congestive heart failure that may result in cardiac transplantation or death [3]. The exact mechanisms of DOX-induced cardiotoxicity are not fully understood but are thought to involve the generation of reactive oxygen species, DNA damage, and mitochondrial dysfunction, making it difficult to predict or prevent its severe adverse effects in patients [4].

In addition to chemotherapy-induced cardiac dysfunction, cancer patients face other comorbidities that can affect their cardiovascular health and overall well-being. Among the various cardiovascular risk factors in cancer patients, psychosocial stress (defined as stress caused by real or perceived unpredictable/uncontrollable life situations that creates an unusual or intense level of adverse social relationships [5]) has emerged as one of the most important and yet understudied factors [6]. Cancer patients often experience a stressful life, stemming from the uncertainty, disease severity, physical difficulties, medical treatments, psychological state, and social relationships-related issues [7]. Stress is considered a major precipitating factor in cancer patients, from diagnosis through treatment, and prognosis, even after the disease is long gone [8]. While the adverse effect of psychosocial stress on cardiovascular functions and cancer-related conditions is well established, animal models that can recapitulate and investigate this association are lacking.

In our previous study, we developed a model in which juvenile mice were exposed to DOX, and psychosocial stress, specifically Chronic Subordination Stress (CSS), was introduced later in adulthood to assess long-term cardiovascular susceptibility to psychosocial stress[9]. Our findings showed that juvenile exposure to DOX exacerbated myocardial fibrosis and inflammation when adult male mice pre-exposed to DOX as juveniles were subjected to chronic subordination stress, highlighting the lasting impact of early-life chemotherapy on cardiac vulnerability under stress [9]. While that model provided important insights into delayed, stress-induced cardiac effects following early DOX exposure, the current study represents a distinct and clinically relevant approach. Here, we investigated the cardiac effects of concurrent exposure to DOX and chronic subordination stress in adult male mice, mimicking a real-world clinical scenario in which cancer patients experience psychosocial stress while actively receiving cardiotoxic chemotherapy. Furthermore, this study expands our understanding of the underlying mechanisms through an unbiased transcriptomic approach to offer a more comprehensive elucidation of how concurrent stress and chemotherapy contribute to cardiotoxicity.

## MATERIALS AND METHODS

### Animal Use and Care

Male C57BL/6N mice, aged 12 weeks, and male previous breeders CD-1 mice were obtained from Charles River Laboratories. The choice of the C57BL/6N sub-strain over C57BL/6J was based on published literature showing that C57BL/6N mice are more susceptible to cardiovascular pathology than C57BL/6J mice [11, 12], and consistency with our previous study [9]. The C57BL/6N mice served as subordinates, while the CD-1 mice were used as dominant, aggressive residents. These stress experiments were run in parallel to our previous study using the same control mice [10] in compliance with the 3R principle. Female laboratory mice do not manifest aggression comparable to male mice, so the CSS model of psychosocial stress is validated only in male mice [13]. Therefore, we only used male mice in this study. Mice were provided ad libitum access to food (2918 Teklad) and water and maintained under 22±2°C and 12:12 hours light: dark. All experimental procedures were approved by the Institutional Animal Care and Use Committee (IACUC) of the University of Minnesota (Protocol 2106–39176A).

### Chronic Subordination Stress (CSS)

Each experimental mouse was paired with a CD-1 mouse for 26 days to establish a stable social hierarchy. Pairing of the two mice remained stable for the entire duration of the study [14, 15]. Social hierarchy was established by interactions occurring daily between 8 and 10 am through direct observation of relevant behaviors, such as upright postures, flight responses, and squeaking vocalizations, as outlined in prior research [14, 15]. Interactions were allowed for a maximum duration of 10 min daily, and pairs were separated if fights escalated to prevent injuries. Mice in the CSS group were housed in standard cages, divided into two equally sized compartments (~620 cm^2^ each) by a plexiglass and wire mesh barrier. This arrangement allowed for continuous sensory exposure (visual, auditory, and olfactory) between experimental and CD1 mice without physical contact, sustaining social stress while preventing injury. In contrast, control mice were individually housed and received daily gentle handling by research staff.

### Study Design and Experimental Groups

**Figure 1:**
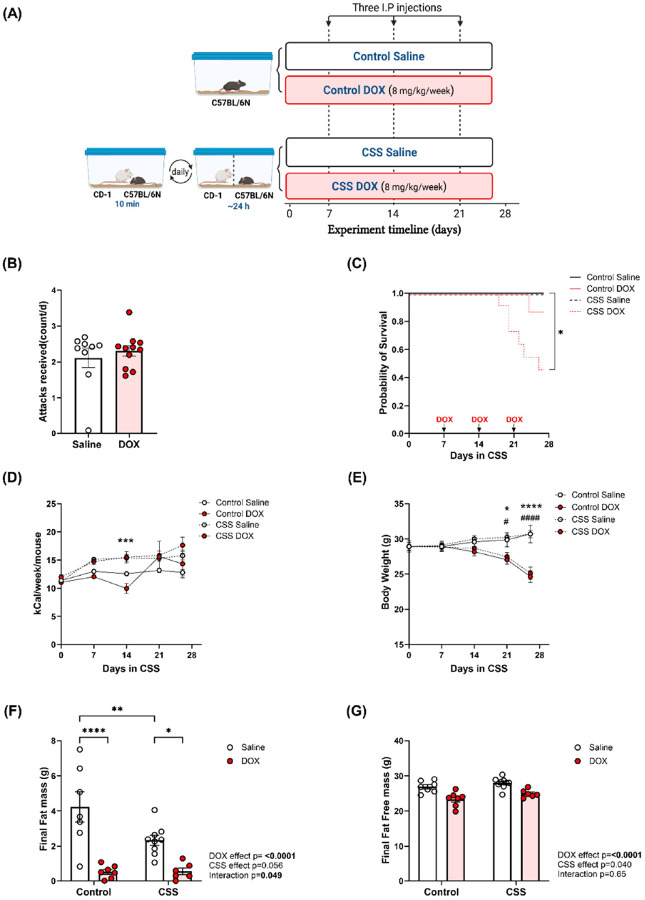
CSS, DOX, and their combined effects on mortality, weight and body composition in adult mice. (A) Experimental design of the study, (B) Average number of attacks received, (C) Kaplan-Meier survival analysis (* indicates a significant difference between CSS Saline and CSS DOX groups), (D) Calorie intake (*indicates a significant difference between Control DOX and CSS DOX groups), (E) Body weights (* within the control group, saline vs DOX; # within the CSS group, saline vs DOX), (F) Final fat mass, and (G) Final fat free mass. Values are represented as means ± SEMs. Statistical analysis was performed by two-way ANOVA with Tukey’s post-hoc analysis as appropriate (*p < 0.05, **p < 0.01, ***p<0.001, ****p < 0.0001). Detailed statistical analysis are provided in Supplemental file 1 Table S2.

The study included four experimental groups (Figure 1A): the **Control Saline** group (n = 7), without CSS and treated with saline; the **Control DOX** group (n = 8), without CSS and treated with DOX; the **CSS Saline** group (n = 9), exposed to CSS and treated with saline (one animal from this group was excluded from echocardiographic data due to outlier ejection fraction and fractional shortening but was included in other analyses); and the **CSS DOX** group (n = 11), combining CSS and DOX (one animal in this group died after echocardiographic data collection but before necropsy, resulting in its exclusion from molecular analyses but inclusion in echocardiographic data. Additionally, due to the lack of PW Doppler images for this animal, it was not included in the diastolic function analysis).

After one week of CSS exposure, mice were randomly assigned to receive one weekly intraperitoneal injection of either 8 mg/kg of DOX or an equivalent volume of sterile saline for three weeks, resulting in a cumulative dose of 24 mg/kg (a dosage regimen equivalent to a human dose of 60–75 mg/m^2^, relevant to clinical doses used in adult cancer patients [16]). At the end of the 26-day CSS exposure, cardiac function was assessed on day 27 through echocardiography. One day post-echocardiography, animals were euthanized in a humane manner via inducing anesthesia with 5% isoflurane followed by decapitation. Hearts were extracted, washed in phosphate-buffered saline, promptly flash-frozen with liquid nitrogen, and stored at −80°C. Food intake was measured weekly, and calorie intake was calculated based on chow weight and caloric content. Data were expressed as kcal per mouse per week.

### Metabolic study and body composition analysis

Fat mass and fat-free mass were assessed using EchoMRI 3-in-1 system from EchoMRI LLC (Houston, TX, USA).

### Echocardiography

Cardiac function was evaluated on the 27th day of the experiment through trans-thoracic echocardiography. Echocardiography was performed using the Vevo 2100 system (Visual Sonics, Inc., Toronto, Ontario, Canada) equipped with an MS400 transducer. Anesthesia was induced with 3% isoflurane and maintained at 1–2% during the procedure. Mice were secured in a supine position on a heated physiologic monitoring stage. Parasternal short axis images of the left ventricle were obtained in M-Mode at the level of the papillary muscles. Endocardial and epicardial borders were manually traced over 3–4 cardiac cycles and parameters of cardiac function such as cardiac output, ejection fraction, left ventricular (LV) mass, fractional shortening, and stroke volume were calculated using Visual Sonics cardiac measurement package of the Vevo 2100. Pulsed wave (PW) Doppler flow images were acquired in the apical 4-chamber view and used to calculate parameters of diastolic function including early (E) and late (A) peak filling blood flow velocities, E-wave deceleration time, isovolumic relaxation time (IVRT), isovolumic contraction time (IVCT), aortic ejection time (AET), and non-filling time (NFT).

### Histopathological analysis

Heart ventricular specimens were obtained, fixed in 10% neutral buffered formalin, and subsequently embedded in paraffin. Sections, measuring four microns in thickness, were subjected to hematoxylin and eosin (HE) or Masson’s trichrome staining to gauge the extent of inflammation or fibrosis. Images were acquired using a Keyence BZ-X810 microscope with a 20x objective, capturing the entire cross-sectional area of the heart tissue. Collagen deposition was quantified based on the area stained in blue, which was measured relative to the total tissue area identified by the red staining. Quantification was performed using CellProfiler software.

### RNA extraction

Total RNA was extracted from 20 mg frozen ventricular tissue using 300 μL Trizol reagent (Thermo Fisher Scientific) according to manufacturer’s instructions. RNA concentrations were calculated by measuring absorbance at 260 nm using a NanoDrop Lite Plus spectrophotometer (Thermo Fisher Scientific).

### Bulk RNA-Seq

Total RNA was extracted and cleaned using the RNeasy Plus micro kit (Qiagen) with genomic DNA depletion. RNA quality was assessed by RiboGreen assay and Agilent BioAnalyzer to ensure >500 ng concentration and RIN ≥8. Libraries were prepared using Illumina’s TruSeq or Stranded mRNA kits, where mRNA was isolated, fragmented, and reverse transcribed to cDNA, followed by adaptor ligation and PCR amplification. Libraries were validated, quantified (PicoGreen, qPCR), normalized, pooled, and size-selected to 320 bp using PippinHT. Sequencing was performed on the Illumina NovaSeq platform with paired-end reads and 2-color SBS chemistry. Base calling and de-multiplexing were done using bcl-convert v4.0.3, generating FASTQ files for downstream analysis (Detailed methods is provided in Supplemental file 1)

### Bioinformatics Analysis

Quality control, data alignment and gene quantification were analyzed using the CHURP pipeline at the University of Minnesota Supercomputing Institute (MSI). 2 × 150bp FASTQ paired-end reads for 16 samples (37.5 million reads average per sample) were trimmed using Trimmomatic (v0.33) enabled with the optional “-q” option; 3bp sliding-window trimming from 3’ end requiring minimum Q30. Quality control on raw sequence data for each sample was performed with FastQC. Read mapping was performed via HISAT2 (v2.1.0) using the mouse genome (GRCm39) as a reference. Gene quantification was done via Feature Counts for raw read counts. Differentially expressed genes (DEGs)were identified using the edgeR (negative binomial, R programming) feature in CHURP using raw read counts. We filtered the generated list based on a minimum log2 fold change [1] and FDR corrected p < 0.05. Volcano plots were generated using the VolcaNoseR website, applying a log2 fold change threshold of >0.5 and a p-value cutoff of <0.01. Gene Set Enrichment Analysis (GSEA) was conducted using R software (version 4.3.0, released 2023-04-21) on a 64-bit x86_64-pc-linux-gnu platform running under Rocky Linux 8.10 (Green Obsidian). The analysis utilized the clusterProfiler package, with hallmark gene sets obtained from the Molecular Signatures Database (MSigDB). The analysis parameters included a user-specified p-value cutoff for DEGs of 0.05 and a log2 fold change (log2FC) cutoff of 1. For ontology analysis, the top 200 DEGs were selected after applying false discovery rate (FDR) and log2FC thresholds. Dot plots were generated in R to visualize the results. For visualization of gene expression patterns, heatmaps were generated using the pheatmap R package (version 1.0.12) in R (version 4.3.1). Genes were separated into upregulated and downregulated groups based on DESeq2 results, with thresholds set at log2 fold change > 0.5 and p < 0.01 for upregulated genes, and log2 fold change < −0.5 and p < 0.01 for downregulated genes. For each group, the top 25 differentially expressed genes were selected by ranking on the absolute log2 fold change. Normalized expression values were log2-transformed and Z-score standardized across genes. Heatmaps were generated using hierarchical clustering on genes (rows) based on Euclidean distance and complete linkage, while samples (columns) were displayed without clustering. Experimental groups were annotated with distinct color labels, and a blue–white–red gradient was used to represent low to high expression levels.

### cDNA synthesis and real-time PCR

First-strand cDNA was synthesized from 1.5 μg total RNA using the Applied Biosystems high-capacity cDNA reverse transcription kit (Thermo Fisher Scientific) according to manufacturer’s instructions. Specific mRNA expression was quantified by SYBR Green-based real-time PCR performed on an Applied Biosystems QuantStudio 5 instrument (Thermo Fisher Scientific) using 384-well optical reaction plates. Thermal cycling parameters were as follows: 95°C for 10 min, followed by 40 PCR cycles of denaturation at 95°C for 15 sec, and annealing/extension at 60°C for 1 min. Gene expression was determined using previously published primers (Supplemental file 1 Table S1). The mRNA expression levels were normalized to β-actin and are expressed relative to the saline-treated controls. Relative gene expression was determined by the ΔΔCT method, and primer specificity and purity of the final PCR product were verified by melting curve analysis.

### Statistical analysis

Data were analyzed using GraphPad Prism software (version 10.1.2, La Jolla, CA) and are presented as means ± standard errors of the mean (SEM). Statistical significance was determined by one- or two-way ANOVA for repeated measures, with Tukey’s HSD post hoc test, or two-way mixed effects ANOVA with Tukey’s post hoc test. A p value of < 0.05 was taken to indicate statistical significance. The specific statistical test used for each experiment is indicated in the figure legends.

## RESULTS

### 1. CSS, DOX, and their combined effects on mortality, weight, and body composition in adult mice

A schematic representation of the study design is illustrated in Fig.1A. As shown in Fig.1B, experimental mice treated with DOX or saline were subjected to a similar amount of aggression by the CD1 male (Fig. 1B). Neither DOX nor CSS alone significantly affected mortality when compared to saline treated Control mice (Fig.1C). However, when DOX was combined with CSS (CSS DOX group), there was a significant increase in mortality (Fig.1C). Caloric intake remained relatively stable in the Control Saline group, progressively increased in CSS Saline and CSS DOX groups and showed a transient reduction in Control DOX group after the first dose of DOX, which was normalized by day 21 (Fig.1D). Body weight trends depicted in Fig.1E revealed stable weight gain in both the Control and CSS saline treated mice, while DOX treatment led to weight loss in both Control and CSS condition. DOX significantly decreased final fat mass (Fig. 1F) compared to saline treatment within both the Control and CSS groups. CSS alone also reduced final fat mass compared to the Control group. In the CSS DOX group, fat mass reduction was comparable to that observed in the group treated with DOX alone, indicating that the combined impact of CSS and DOX did not further enhance fat loss beyond DOX treatment alone (Supplemental file 1 Fig. 1 represents fat mass trajectory comparing baseline with CSS). Final fat-free mass was not affected by DOX or CSS either alone or in combination (Fig.1G and Supplemental file 1 Fig. 2). Overall, these results demonstrate that DOX treatment induced significant weight loss, and reductions in fat mass. When DOX was combined with CSS it also caused an increase in mortality.

### 2. CSS, DOX, and their combined effects on cardiac function in adult mice

**Figure 2:**
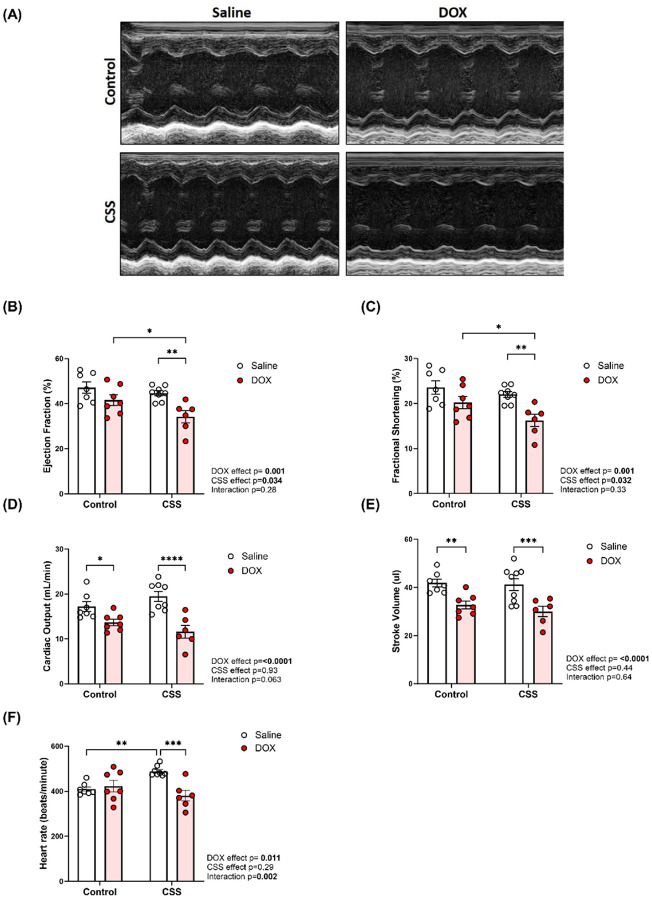
CSS, DOX, and their combined effects on cardiac function in adult mice. (A) Representative M-Mode images of parasternal short axis view of the heart, (B) Ejection fraction, (C) Fractional shortening, (D) Cardiac output, (E) Stroke volume, and (F) Heart rate. Values are represented as means ± SEMs (n=7–11 per group). Statistical analysis was performed by two-way ANOVA with Tukey’s post-hoc analysis as appropriate (*p<0.05, **p<0.01, ***p<0.001, ****p<0.0001). Detailed statistical analysis are provided in Supplemental file 1 Table S3.

Trans-thoracic echocardiography was performed at the end of the experiment to evaluate the effects of DOX, CSS, and their combined influence on cardiac function and morphology. Representative echocardiographic images obtained in M-Mode from each group are displayed in Fig. 2A. The analysis of cardiac function demonstrated that DOX alone did not significantly alter ejection fraction (EF) (Fig. 2B) or fractional shortening (FS) (Fig. 2C). However, DOX significantly reduced cardiac output (CO) (Fig. 2D) and stroke volume (SV) (Fig. 2E). CSS alone had no significant effects on these parameters but significantly elevated heart rate (HR) (Fig. 2F). When DOX was combined with CSS, a marked decline in EF (Fig. 2B) and FS (Fig. 2C) was observed, indicating systolic dysfunction. Additionally, the CSS-induced increase in HR was abrogated by concurrent DOX administration (Fig. 2F). Collectively, these findings highlight that DOX and CSS have distinct effects on cardiac function, but their combined exposure exacerbates systolic dysfunction.

### 3. CSS, DOX, and their combined effects on cardiac diastolic function in adult mice

**Figure 3:**
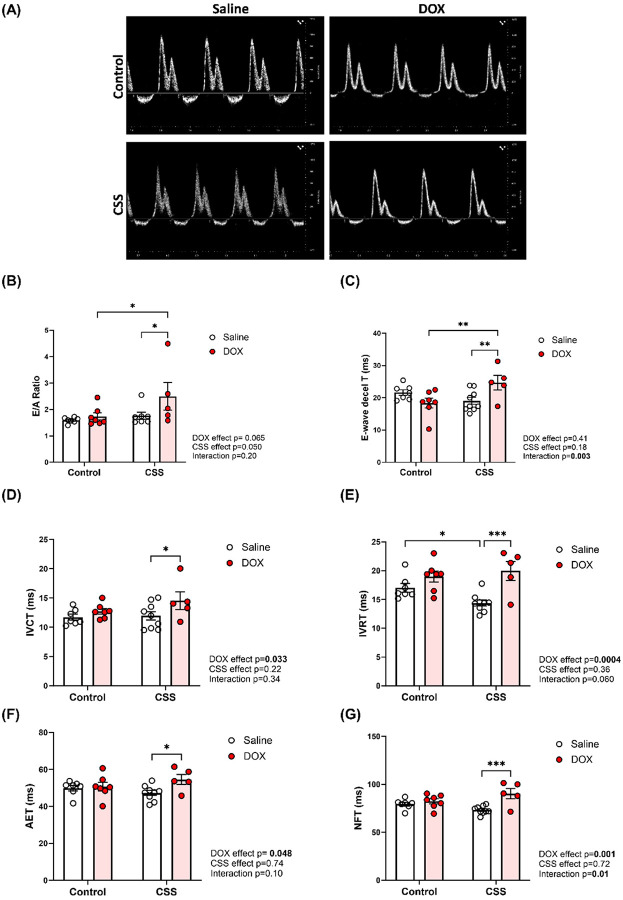
CSS, DOX, and their combined effects on cardiac diastolic function in adult mice. (A) Representative images of the apical 4-chamber view, (B) E/A ratio, (C) E-wave deceleration time, (D) Isovolumic contraction time (IVCT), (E) Isovolumic relaxation time (IVRT), (F) Aortic ejection time (AET), and (G) Non-flow time (NFT). Values are represented as means ± SEMs (n=7–11 per group). Statistical analysis was performed by two-way ANOVA with Tukey’s post-hoc analysis as appropriate (*p<0.05, **p<0.01, ***p<0.001). Detailed statistical analysis are provided in Supplemental file 1 Table S3.

Diastolic function was assessed using images obtained in the apical 4-chamber view by transthoracic echocardiography (Fig. 3A). The E/A ratio and E-wave deceleration time did not differ significantly between saline-treated controls, DOX-treated controls, and CSS-saline mice. However, mice subjected to the combined CSS and DOX treatment exhibited a significant elevation in both the E/A ratio and E-wave deceleration time when compared to other experimental groups (Fig. 3B, 3C), indicating altered ventricular filling dynamics. Moreover, key time intervals associated with diastolic function-namely, isovolumic contraction time (IVCT), isovolumic relaxation time (IVRT), aortic ejection time (AET), and non-flow time (NFT)-were significantly prolonged in the CSS DOX group relative to other experimental groups (Fig. 3D–3G). The observed diastolic abnormalities in the CSS DOX group suggest an additive/synergistic deleterious effect of CSS and DOX exposure on left ventricular diastolic function.

### 4. CSS, DOX, and their combined effects on cardiac morphometry in adult mice

**Figure 4:**
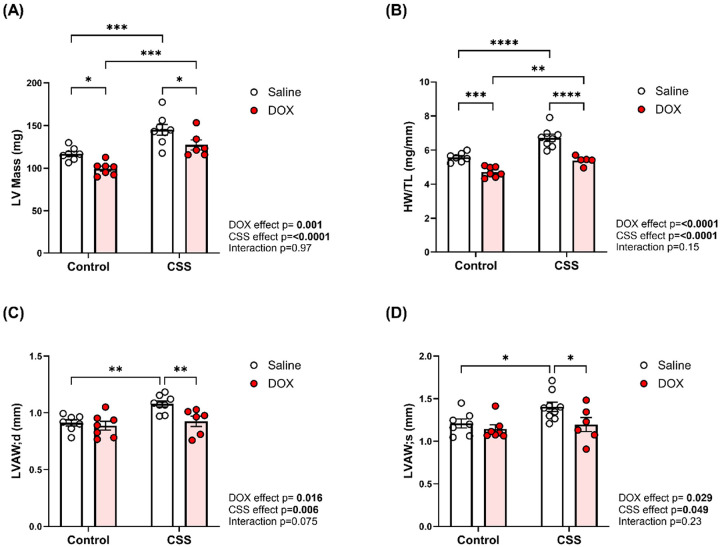
CSS, DOX, and their combined effects on cardiac morphometry in adult mice. (A) Left ventricular mass, (B) Heart weight/tibia length (HW/TL), (C) left ventricular anterior wall thickness at diastole (LVAW;d), (D) left ventricular anterior wall thickness at systole (LVAW;s). Values are represented as means ± SEMs (n=7–11 per group). Statistical analysis was performed by two-way ANOVA with Tukey’s post-hoc analysis as appropriate (*p<0.05, **p<0.01, ***p<0.001, ****p<0.0001). Detailed statistical analysis are provided in Supplemental file 1 Table S3.

Cardiac morphometry revealed that DOX induced LV atrophy, evidenced by reduced LV mass (Fig. 4A), and heart weight-to-tibia length ratio HW/TL (Fig. 4B), while CSS alone caused hypertrophy. Combined treatment attenuated the CSS-induced hypertrophy, indicating that DOX can counterbalance the hypertrophic pathways activated by CSS. Structural analysis of the left ventricular anterior wall thickness at end diastole (LVAW;d) and end systole (LVAW;s) showed a significant increase under CSS alone (Fig. 4C and 4D, respectively). While posterior wall thickness at end diastole (LVPW;d) was unaffected by DOX alone, it significantly increased under CSS in the presence of DOX. However, no differences were observed in posterior wall thickness during systole (LVPW; s) across groups (Supplemental Table 3). Additionally, left ventricular internal diameters (LVID;d and LVID;s) and volumes (LV Vol;d and LV Vol;s) remained unchanged (Supplemental file 1 Table 3), indicating preserved chamber dimensions despite the observed wall remodeling.

### 5. CSS, DOX, and their combined effects on cardiac fibrosis and markers of cardiotoxicity in adult mice

**Figure 5:**
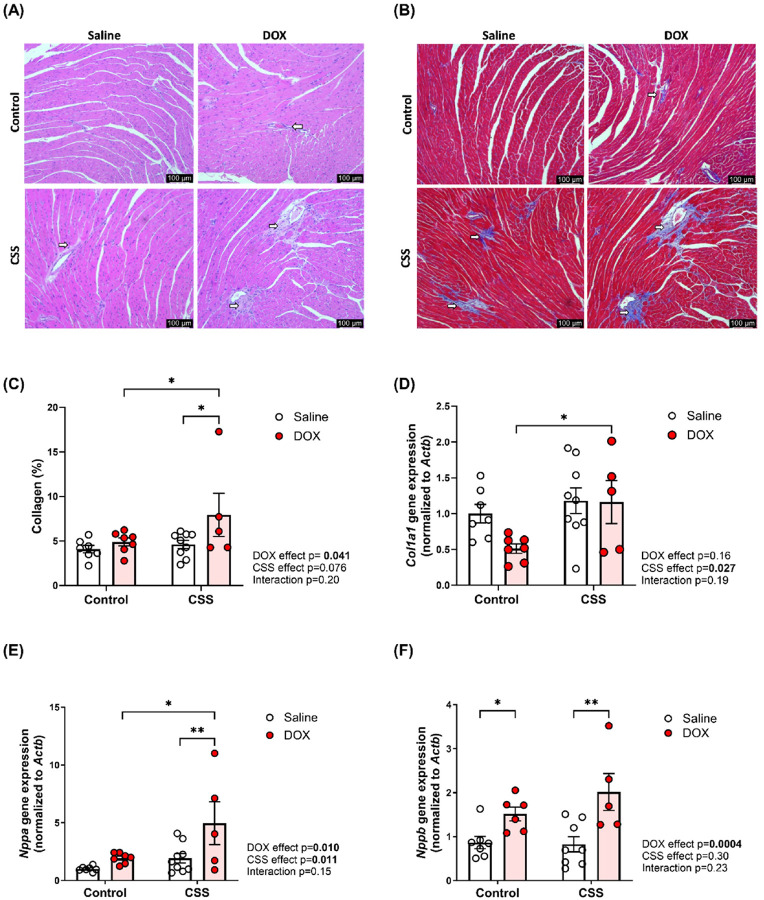
CSS, DOX, and their combined effects on cardiac fibrosis and cardiac stress response in adult mice. Representative images from (A) Hematoxylin and eosin and (B) Masson’s trichrome stained left ventricular heart sections, bar scale= 100μm. Inflammatory cell infiltration in (A) and fibrotic areas in (B) are indicated with arrows. (C) Collagen content (%), (D) The mRNA expression of *Collagen 1a1* (*Col1a1*), (E) *Natriuretic peptide A* (*Nppa*), and (F) *Natriuretic peptide B* (*Nppb*). Values are represented as means ± SEMs (n=5–9 per group). Statistical significance of pairwise comparisons was determined by two-way ANOVA with Tukey’s post-hoc analysis as appropriate (*p<0.05, **p<0.01). Detailed statistical analyses are provided in Supplemental file 1 Table S4.

Histopathological analysis of left ventricular heart tissue using hematoxylin and eosin (HE) (Fig. 5A) staining and Masson’s trichrome staining (Fig. 5B) revealed evidence of inflammation and fibrosis when CSS is combined with DOX. Notably, quantitative assessment of collagen content (%) revealed a significant elevation in DOX-treated mice subjected to CSS compared to both DOX-treated mice without CSS and CSS-exposed mice receiving saline, indicating a potentiated fibrotic response under combined CSS and DOX insult (Fig. 5C). Gene expression analysis further supported these findings, showing upregulation of *Col1a1*, a key marker of extracellular matrix remodeling and fibrosis, caused by CSS as a main factor, with a significant increase in mice exposed to DOX and CSS (Fig. 5D). Additional gene expression analysis revealed significant upregulation of *Nppa*, a gene associated with cardiac pathologic remodeling, in DOX-treated mice exposed to CSS, with both CSS and DOX as driving factors (Fig. 5E). Finally, the expression of *Nppb*, another critical marker of pathologic remodeling, was significantly and mainly elevated by DOX treatment in both control and CSS groups, while CSS alone had no effect (Fig. 5F). These findings demonstrate that CSS is a key contributor to cardiac inflammatory and fibrotic responses, while DOX drives the induction of markers of cardiotoxicity (*Nppa* and *Nppb*).

### 6. Bulk RNA-Seq analysis uncovers cardiac stress and injury pathways induced by CSS and DOX

**Figure 6:**
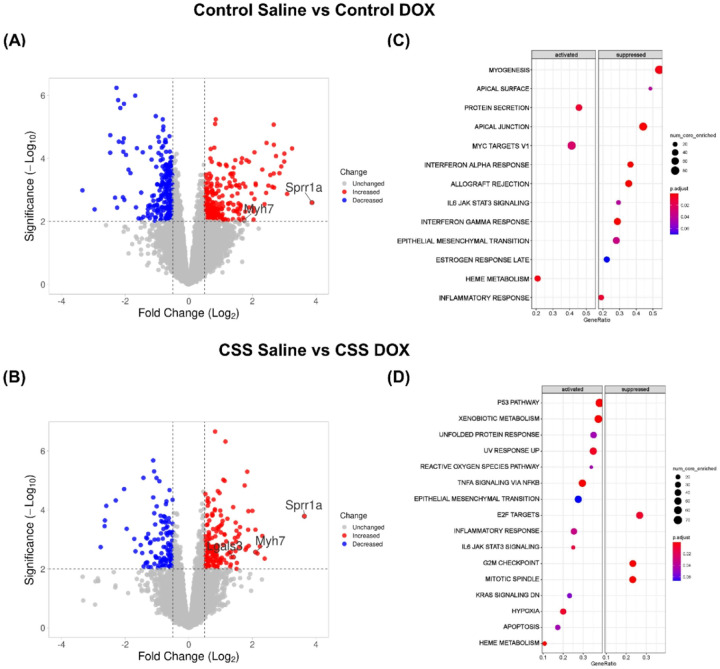
Bulk RNA-Seq Analysis of DOX treatment, CSS exposure, and their combined effects. Volcano plot of the DEGs in (A) Control Saline vs Control DOX, (B) Control Saline vs CSS Saline, and (C) CSS Saline vs CSS DOX. Dot plots representing Gene GSEA comparing(D) Control Saline vs Control DOX, (E) Control Saline vs CSS Saline, and (F) CSS Saline vs CSS DOX. The y-axis lists hallmark gene sets from the MSigDB. The x-axis represents the GeneRatio, defined as the fraction of genes from the pathway that overlap with the input gene set relative to the total number of genes in the pathway. The size of each dot (num_core_enriched) reflects the number of core-enriched genes contributing to the pathway enrichment, with larger dots indicating a greater number of enriched genes. The color of each dot (p.adjust) corresponds to the adjusted p-value of enrichment (FDR-adjusted p-value), with red representing lower p-values (higher significance) and blue indicating higher p-values (lower significance). Detailed information is provided in Supplemental file 2 Sheets 3–4.

To determine the transcriptional changes associated with DOX treatment, CSS exposure, and their combined effects, bulk RNA sequencing was conducted on heart tissue samples from study mice (n = 4 per group). We analyzed differential gene expression across the experimental comparisons: Control Saline vs. Control DOX (Fig. 6A), and CSS Saline vs. CSS DOX (Fig. 6B). Volcano plots (Fig. 6A–B) illustrate significantly upregulated (red) and downregulated (blue) DEGs in each condition. Based on these criteria, the comparison between Control DOX vs Control saline groups revealed 105 upregulated and 42 downregulated DEGs (Supplemental file 2 Sheet1). Similarly, in the CSS DOX vs. CSS Saline comparison, 44 genes were upregulated, and 63 genes were downregulated (Supplemental file 2 Sheet2). A subset of cardiac-relevant genes including *Myh7*, *Sprr1a*, and *Lgals3*, were annotated in the volcano plots.

To gain further insight into the biological pathways affected by these conditions, we performed GSEA to identify activated and suppressed pathways in each comparison (Fig. 6C–D). In the Control Saline vs Control DOX comparison (Fig. 6C), key pathways related to myogenesis, and inflammatory responses were significantly suppressed, consistent with DOX known effect on muscle degeneration, and immune modulation. Conversely, the upregulated pathways, although significantly enriched, were not directly associated with cardiotoxicity-related mechanisms. In parallel, heatmap analyses of the top 25 upregulated and downregulated DEGs were performed for this comparison, providing a visual overview of the most prominent gene expression changes (Supplemental File 1, Fig. 3A-B). The CSS Saline vs CSS DOX comparison (Fig. 6D) exhibited an enrichment of pathways related to p53 signaling, TNFα signaling via NFκB, apoptosis, IL6/JAK/STAT signaling and hypoxia, suggesting an exacerbated cellular stress response. The activation of pro-apoptotic and inflammatory signaling suggests that CSS exposure, in combination with DOX, may contribute to increased oxidative stress, inflammatory cytokine signaling, and hypoxia-related transcriptional programs. Corresponding heatmap analyses of DEGs for this comparison also supported these findings by highlighting distinct expression patterns (Supplemental File 1, Fig. 4A-B). Meanwhile, pathways associated with cell cycle regulation, such as G2M Checkpoint and Mitotic Spindle, were suppressed, suggesting impaired cell cycle progression and senescence. (Detailed information is provided in Supplemental file 2 Sheets 3–4).

### 7. Validating differentially expressed genes by quantitative real-time PCR

**Figure 7:**
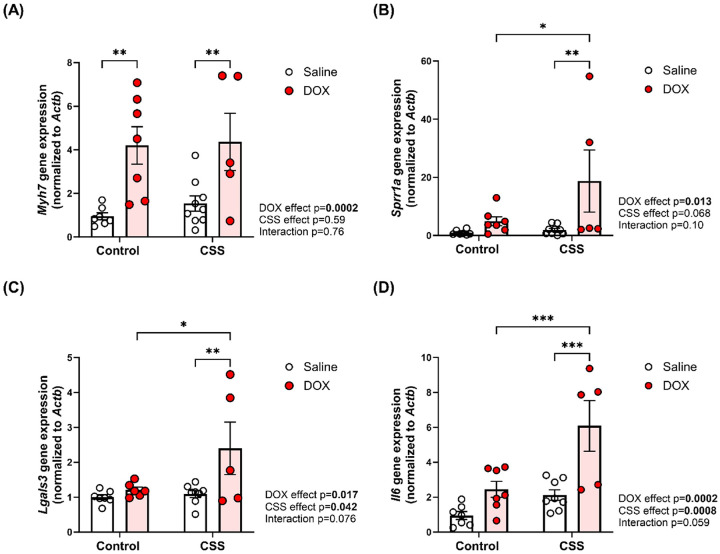
Validation of DEGs by real-time PCR. The mRNA expression of (A) *Myosin heavy chain 7* (*Myh7*), (B) *Small proline-rich protein 1A* (*Sprr1a)*, (C) *Galectin-3* (*Lgals3*), and (D) *Interleukin-6* (*Il-6*). Values are represented as means ± SEMs. n = 5–9 per group. Statistical significance of pairwise comparisons was determined by two-way ANOVA with Tukey’s post-hoc analysis as appropriate (*p<0.05, **p<0.01, ***p<0.001).

To validate the differential gene expression results of genes with cardiac relevance, quantitative real-time PCR was performed on selected genes of interest. Real-time PCR confirmed significant transcriptional changes induced by DOX and CSS. *Myh7*, the gene encoding β-myosin heavy chain, was significantly upregulated in response to DOX in both control and CSS groups (Fig. 7A). Myh7 is a well-established marker of pathologic cardiac remodeling. *Sprr1a*, a gene associated with pro-apoptotic/pro-fibrotic remodeling, and *Lgals3*, a marker of fibrosis and inflammation, were both significantly upregulated in the CSS DOX group (Fig. 7B and 7C, respectively). Furthermore, the pro-inflammatory cytokine *Il-6*, was significantly upregulated in the CSS DOX group (Fig. 7D). This observation aligns with the GSEA results (Fig. 6F), which identified inflammation-related pathways, including the IL6-JAK-STAT signaling, as a key component of the transcriptional changes induced by DOX in CSS-exposed mice. Together, these findings underscore the roles of fibrosis and inflammation in the exacerbation of DOX-induced cardiotoxicity when compounded by CSS.

## DISCUSSION

Doxorubicin, though effective against cancer, is limited by cardiotoxic effects driven by oxidative stress, mitochondrial dysfunction, and cardiomyocyte apoptosis [17–19]. Adding to the cardiotoxic burden, cancer patients often experience psychosocial stress, anxiety, and depression, which are exacerbated during chemotherapy and negatively affect quality of life and treatment adherence [20, 21]. In turn, psychosocial stress is a risk factor for CVD with a magnitude comparable to traditional risk factors such as smoking and obesity [22]. Despite its clinical relevance, no preclinical models exist to examine the concurrent impact of DOX and psychosocial stress on cardiovascular health in adult mice. Here, we filled this knowledge gap by developing a mouse model in which a chronic stress protocol (i.e. CSS) is combined with DOX treatment in adult mice. The combined CSS/DOX caused cardiac dysfunction, increased markers of cardiotoxicity, inflammation, fibrosis, and cell cycle inhibition as well as increased mortality rate.

Previous studies suggest that psychosocial stress exacerbates the cardiotoxic effects of chemotherapy drugs like DOX by increasing oxidative stress, inflammation, and sympathetic nervous system activation [9, 21]. DOX-induced heart failure (HF) has a 5-year survival rate of less than 50%, significantly worse than idiopathic or ischemic cardiomyopathy, with cancer patients experiencing DOX-induced HF having even poorer outcomes [23, 24]. Our study builds on this by showing that combining DOX with CSS increased the mortality rate to > 50%. This significant escalation aligns with findings from a murine model of arrhythmogenic cardiomyopathy, which provided evidence that psychosocial stress (a resident-intruder model similar to CSS), increases the risk of sudden cardiac death [25], and with epidemiological studies linking low socioeconomic status to increased morbidity and reduced lifespan in humans [26–28].

Our data show that DOX alone causes marked cardiac atrophy as indicated by a reduction in LV mass and HW/TL, without significantly altering EF or FS. By contrast, CSS alone induces hypertrophy without changing systolic indices. Strikingly, the CSS DOX group exhibits both systolic and diastolic dysfunction. While previous studies have independently shown that DOX impairs both systolic and diastolic function in rodent models [29], and that psychosocial stress, particularly when combined with metabolic comorbidities such as obesity[30], can lead to similar cardiac dysfunction, no prior studies have explicitly examined the combined effects of DOX and CSS on cardiac function, underscoring the novelty of the present work. Notably, DOX blunted the CSS-induced hypertrophy. Wall thickness in the combined group are normalized despite unaltered chamber dimensions, underscoring that CSS and DOX activate opposing remodeling programs. Together, these results indicate a maladaptive interaction between CSS-driven hypertrophy and DOX-driven atrophy, yielding combined structural remodeling, and systolic and diastolic dysfunction [31, 32].

*Nppa* and *Nppb* are well-established markers of cardiotoxicity, with their expression known to increase under various pathological conditions, including DOX-induced injury [33, 34]. In our study, *Nppa* was significantly upregulated only as a consequence of dual exposure with DOX and CSS. *Nppb*, another key indicator of cardiac stress, was significantly induced by DOX in both control and CSS-exposed mice [35], suggesting that *Nppb* is predominantly driven by DOX-induced cardiotoxicity rather than CSS. These findings underscore the distinct contributions of DOX to stress-related pathways and maladaptive cardiac remodeling, while CSS is involved in specific aspects of the cardiac stress response. Consistent with the previous studies showing that CSS induces cardiac fibrosis [25], our study identified significant fibrotic remodeling in the CSS DOX group, with much of this attributed to the effects of CSS alone. Lastly, our analysis revealed increased collagen deposition in the CSS DOX group, indicative of extensive extracellular matrix remodeling, which may be mediated by *Col1a1*.

To identify underlying molecular mechanisms, bulk RNA-seq was conducted on heart tissue samples from mice. Differential gene expression analysis revealed substantial transcriptional alterations induced by DOX alone, CSS alone, or their combination. The RNAseq analysis revealed a number of DEGs with known connections to cardiac pathologic remodeling, including *Lgals3*, *Myh7*, and *Sprr1a*. *Lgals3*, a pivotal mediator of fibrosis and inflammation, plays a critical role in extracellular matrix remodeling and cardiac dysfunction [36]. In our study, elevated *Lgals3* expression was strongly associated with fibrotic remodeling induced by the combined CSS and DOX. This upregulation was corroborated by increased *Col1a1* expression and a higher percentage of collagen deposition, collectively indicating pronounced cardiac fibrosis. This can be associated with reductions in EF and FS, highlighting the role of *Lgals3*-driven remodeling in systolic dysfunction [37]. *Myh7*, a fetal gene re-expressed during pathological cardiac remodeling and heart failure [35], was significantly upregulated by DOX independent of CSS exposure. These findings highlight the distinct contributions of DOX to stress-related pathways and maladaptive cardiac remodeling. A novel finding of this study was the significant upregulation of *Sprr1a* in the CSS DOX group-a gene previously implicated in maladaptive cardiac remodeling, fibroblast activation, and apoptosis under pathological conditions [38]. These findings highlight Sprr1a as a potential molecular driver of the pathological remodeling observed in this dual-stressor model.

GSEA provided deeper insights into the molecular mechanisms underlying these observations. In the Control Saline vs Control DOX comparison, pathways associated with protein synthesis, metabolism, and cellular growth were significantly activated in the DOX-treated group. In contrast, pathways linked to immune signaling and inflammatory response were suppressed, indicating a disruption in immune homeostasis due to DOX exposure in control mice. In contrast, the CSS Saline vs. CSS DOX comparison revealed robust activation of pathways associated with inflammation, apoptosis, and tissue remodeling, alongside suppression of cell cycle–related pathways, suggesting impaired regenerative potential in response to the combined insult of CSS and DOX. Notably, strong activation of the inflammatory response and IL6-JAK-STAT3 signaling in the CSS DOX group was consistent with histological findings of increased inflammation and was supported by elevated expression of *Il6*, a key pro-inflammatory cytokine implicated in cardiac fibrosis and dysfunction[39]. These findings align with our previously published work demonstrating that psychosocial stress is associated with heightened systemic inflammation in cancer survivors, further supporting the translational relevance of the current findings [40].

### Study Limitations

First, while the CSS DOX group showed significantly increased mortality, the exact cause remains unclear. Although cardiac dysfunction was observed, other contributors such as arrhythmias, hypertension, or autonomic dysregulation cannot be ruled out as a cause for increased mortality. Second, the study was limited to male mice, as the CSS model is currently validated only in males [41]. Third, echocardiography data show cardiac function and morphometry at a single endpoint. Longitudinal imaging would have provided better insight into the progression and timing of cardiac dysfunction and will be included in future investigations.

In conclusion, this study is the first to establish a preclinical model demonstrating the synergistic cardiotoxic effects of CSS and DOX in adult mice. The combined CSS DOX treatment led to significantly higher mortality, systolic and diastolic dysfunction, and myocardial remodeling characterized by inflammation and fibrosis. Transcriptomic and histological analyses revealed activation of pro-inflammatory and pro-fibrotic pathways, including IL6-JAK-STAT3 signaling, as well as increased expression of genes related to pathological cardiac remodeling such as *Lgals3, Sprr1a*, and *Il6*. Together, these findings highlight the critical role of psychological stress in exacerbating DOX-induced cardiotoxicity and underscore the need for integrative cardio-oncology approaches that consider both pharmacological and psychosocial contributors to cardiac risk in cancer patients.

## Supplementary Material

Supplementary Files

This is a list of supplementary files associated with this preprint. Click to download.

- SUPPLEMENTALMATERIAL1.docx
- SUPPLEMENTALMATERIAL2.xlsx

## Data Availability

Data will be made available upon reasonable request.
